# Effects of mesenchymal stromal cells versus serum on tendon healing in a controlled experimental trial in an equine model

**DOI:** 10.1186/s12891-018-2163-y

**Published:** 2018-07-18

**Authors:** A. B. Ahrberg, C. Horstmeier, D. Berner, W. Brehm, C. Gittel, A. Hillmann, C. Josten, G. Rossi, S. Schubert, K. Winter, J. Burk

**Affiliations:** 1 Department of Orthopedics, Traumatology and Plastic Surgery, University of Leipzig, Liebigstr. 20, 04103 Leipzig, Germany; 2 Translational Center for Regenerative Medicine (TRM), University of Leipzig, Leipzig, Germany; 3 Saxon Incubator for Clinical Translation (SIKT), University of Leipzig, Leipzig, Germany; 4 University Equine Hospital, University of Leipzig, Leipzig, Germany; 5 Department of Clinical Science and Services, The Royal Veterinary College, University of London, London, UK; 6 School of Biosciences and Veterinary Medicine, University of Camerino, Camerino, Italy; 7 Institute of Veterinary Physiology, University of Leipzig, Leipzig, Germany; 8 Institute of Anatomy, Medical Faculty, University of Leipzig, Leipzig, Germany; 9 Department of Biotechnology, University of Natural Resources and Life Sciences, Vienna, Austria

**Keywords:** MSC, Serum, Animal model, Tendon, Horse

## Abstract

**Background:**

Mesenchymal stromal cells (MSC) have shown promising results in the treatment of tendinopathy in equine medicine, making this therapeutic approach seem favorable for translation to human medicine. Having demonstrated that MSC engraft within the tendon lesions after local injection in an equine model, we hypothesized that they would improve tendon healing superior to serum injection alone.

**Methods:**

Quadrilateral tendon lesions were induced in six horses by mechanical tissue disruption combined with collagenase application 3 weeks before treatment. Adipose-derived MSC suspended in serum or serum alone were then injected intralesionally. Clinical examinations, ultrasound and magnetic resonance imaging were performed over 24 weeks. Tendon biopsies for histological assessment were taken from the hindlimbs 3 weeks after treatment. Horses were sacrificed after 24 weeks and forelimb tendons were subjected to macroscopic and histological examination as well as analysis of musculoskeletal marker expression.

**Results:**

Tendons injected with MSC showed a transient increase in inflammation and lesion size, as indicated by clinical and imaging parameters between week 3 and 6 (*p* < 0.05). Thereafter, symptoms decreased in both groups and, except that in MSC-treated tendons, mean lesion signal intensity as seen in T2w magnetic resonance imaging and cellularity as seen in the histology (p < 0.05) were lower, no major differences could be found at week 24.

**Conclusions:**

These data suggest that MSC have influenced the inflammatory reaction in a way not described in tendinopathy studies before. However, at the endpoint of the current study, 24 weeks after treatment, no distinct improvement was observed in MSC-treated tendons compared to the serum-injected controls. Future studies are necessary to elucidate whether and under which conditions MSC are beneficial for tendon healing before translation into human medicine.

## Background

In orthopedic surgery and sports medicine, tendinopathy has become one of the most challenging conditions. Achilles tendinopathy, for example, is a growing problem with a high incidence [1]. Various therapies have been tried without lasting success [2–8]. This underlines the need for a new curative therapy from the field of regenerative medicine. The horse, which could be referred to as animal athlete, suffers from a natural tendinopathy in its superficial digital flexor tendon (SDFT) which is similar to the Achilles tendon in its structure, function and pathophysiology [9–12]. Therefore, horses are considered as highly suitable model animals for orthopedic research and have been recommended by authorities such as the U.S. Food and Drug Administration (FDA) and the European Medicines Agency (EMA) [13, 14]. Contrary to human medicine, regenerative cell therapies have already been used in equine medicine for more than a decade, with most experiences in multipotent mesenchymal stromal cells (MSC) therapy for tendon disease [15]. Clinical results are promising, although the mechanisms of action leading to improved tendon healing are not yet fully understood [11, 16, 17].

MSC are adult progenitor cells displaying plastic-adherence and multipotent differentiation potential in vitro and can be further characterized by a set of surface marker antigens [18]. They can be extracted from various tissues, e.G. adipose tissue or bone marrow and are thought to support tissue regeneration not only by cell replacement, but also via trophic and modulatory effects which are mediated by cell-cell contact or paracrine mechanisms [19–21].

The promising results of MSC therapy in equine patients make a translation into human medicine appear reasonable. Recently we were able to show that adipose-derived MSC engrafted within tendon lesions after local injection in an equine model although there was also systemic distribution of a small number of cells [22]. These results were obtained on the basis of labeling the MSC with superparamagnetic iron oxide (SPIO) and rhodamine. After demonstrating long-term MSC persistence we aimed to evaluate the effect of these MSC on tendon healing within the same set of animals as presented in the current study. We hypothesized that local injection of adipose-derived MSC improves outcome parameters obtained by clinical imaging and histological assessment as well as gene expression analysis over a follow-up period of 24 weeks.

## Methods

### Study design

Tendinopathy of the SDFT was induced in all limbs of 6 horses and autologous adipose tissue was harvested (week − 3). MSC were expanded and labeled with SPIO. MSC resuspended in serum or serum alone were injected into the left and right tendon lesions 3 weeks after their induction (week 0). Clinical examinations and diagnostic imaging were performed over 24 weeks (week 0, 1, 2, 3, 4, 6, 8, 12 and 24). Tendon biopsies were taken after 3 weeks from the hindlimb SDFT for early histological evaluation (week 3). After euthanasia at week 24, forelimb SDFT were harvested for macroscopic and late histological evaluation and for analysis of musculoskeletal marker expression. Approval of the local ethics committee (TVV 34/13) had been given beforehand.

### Animals

Six horses (standardbred; mean age: 6 years; age range: 3–10 years; 3 female, 3 male; body weight: 400–550 kg) were included in the study. The horses were obtained from trot racing courts in accordance with the local authority. Clinical evaluation, ultrasound (US) and Magnetic Resonance Imaging (MRI) of all extremities were performed prior to the first surgery to ensure the animals were healthy. The horses were housed in the hospital’s stables. All procedures were performed within the facility, so no transport of the horses was needed.

### Induction of tendinopathy and adipose tissue collection

Tendinopathy was induced by minor mechanical tissue disruption combined with a low-dose injection of collagenase type I (4.8 mg/mL, Life Technologies GmbH, Darmstadt, Germany) in the SDFT of all 4 extremities under general anesthesia (week − 3). With the horse placed in lateral position, the skin was clipped and prepared aseptically. An 11-gauge bone marrow aspiration needle was introduced into the SDFT in the middle of the metatarsal/metacarpal region via a skin incision. 0.4 mL collagenase type I (250 IU per tendon lesion) were injected while removing the needle from 2 cm proximal to 1 cm proximal from its entry point without further damage of the epitenon. The incisions in the peritendineum and skin were closed by suture and a dressing was applied. Subcutaneous adipose tissue was obtained from the supragluteal region of the horse in the same surgery. The same orthopedic surgeon performed all surgeries.

### Post-surgery regime

After induction of tendinopathy, horses were restricted to stall rest for 5 weeks (2 weeks after MSC injection) and then managed according to the rehabilitation protocol previously described [23].

The horses were checked 3 times a day using standardized pain scores until 10 days post-surgery. For pain relief, they received flunixin-meglumine (CP-Pharma Handelsgesellschaft mbH, Burgdorf, Germany), 1.1 mg/kg bwt twice daily on the day of surgery and 0.55 mg/kg bwt twice daily on day 1 to 4 post surgery, followed by 0.55 mg/kg once daily on day 5 and 6, as well as additional single 1.1 mg/kg bwt doses when required according to pain scoring results.

### Cell isolation and injection

The adipose tissue was subjected to cell isolation by collagenase digestion and plastic-adherent cells were isolated and expanded as described previously [24]. The cells were cultivated in a standard culture medium with Dulbecco’s Modified Eagle’s Medium (DMEM, Life Technologies GmbH, Darmstadt, Germany), 20% Fetal Bovine Serum (FBS, Sigma-Aldrich Chemie GmbH, Steinheim, Germany), 1% Penicillin (10.000 IE/ml)/Streptomycin (10.000 μg/ml, Life Technologies GmbH, Darmstadt, Germany) and 0,1% Gentamicin (50 mg/ml, Life Technologies GmbH, Darmstadt, Germany). At passage 2 and 80% confluency, MSC were labeled with SPIO particles (Molday ION Rhodamine B™, BioPAL, Inc.™, Worcester, MA, USA) at 25 μg Fe per ml for 20 h and harvested. Part of the cells from each animal was used to confirm MSC characteristics such as trilineage differentiation and expression of CD29, CD44, CD90 and CD105 as described before [24, 25].

Three weeks after lesion induction (week 0), the freshly harvested and labeled MSC were injected into the lesions under ultrasonographic guidance. Horses were sedated and received an ulnar nerve block (forelimbs) or a high plantar nerve block (hindlimbs) with additional local anesthesia (Lidocain 2%, bela-pharm GmbH & Ko. KG, Vechta, Germany). With the horses standing, the skin was clipped and prepared aseptically. In a randomized manner, one hindlimb and one forelimb were injected with MSC (10^7^ cells in 1 ml autologous serum) and the contralateral side with 1 ml autologous serum. All injections were performed in a standardized procedure by the same veterinary surgeon blinded to the content of the syringes.

### Clinical and imaging evaluation

Two veterinarians blinded for the treatment performed all examinations in a standardized procedure.

Clinical parameters comprised a palpation score and a lameness score. The palpation score included diffuse and local swelling, heat and pain to palpation, the total score points ranging from 4 (normal) to 16 (severe). The lameness score included weight bearing/lameness when standing, walking, trotting, and turning, the total score points ranging from 7 (normal) to 29 (severe).

Ultrasonographic examinations of the SDFT were performed using a 10 MHz linear transducer (LOGIQ 5 Expert, GE Healthcare, Munich, Germany) with a standoff probe. The SDFT was divided into zones and at least one transverse and one longitudinal image was recorded at every level [26]. Echogenicity of the lesion, peritendinous edema and fiber pattern were evaluated for each zone by 2 blinded observers in consensus, using a semi quantitative score [27]. Furthermore, the cross-sectional areas of the tendon lesion and the whole tendon were measured in each transverse image and used to calculate the percentage of the tendon lesion.

MRI of the SDFT region was performed in the standing sedated horse using a 0.27 Tesla dedicated equine low-field MRI system (Hallmarq EQ2, Hallmarq Veterinary Imaging, Guildford, Surrey, UK). T2-weighted (T2w) fast spin echo sequences acquired in transverse plane at weeks 0, 3, 12 and 24 were used to determine lesion signal and lesion areas. Lesion volume was approximated by multiplying the sum of the lesion areas in all images by 6 mm (5 mm slice thickness + 1 mm gap). Image processing was performed using Mathematica (Wolfram Research, Inc., Mathematica, Version 10.3.0.0, Champaign, IL). Tendon regions were manually drawn onto the images and converted into binary image masks. Background-corrected MRI images were segmented, and segmentations were multiplied with the corresponding binary image masks to detect lesion areas within the tendons. Positive regions in the resulting binary images were smoothed and final binary lesion masks were obtained. Grayscale values for tendon and lesion regions were extracted from the background-corrected images using the generated masks.

For all parameters obtained from ultrasonographic images as well as lesion signal intensity obtained from MRI images, mean values of images obtained from the different tendon levels were calculated and used for further analysis, in order to account for the proximo-distal dimensions of the lesions.

### Collection of tendon samples

Three weeks after MSC injection, tendon biopsies of the maximum lesion, which had been identified by MRI before, were taken from the hindlimbs under general anesthesia. The tendon was approached via a skin incision of 3 cm and 0.2 × 0.3 × 2 cm tendon tissue was collected and fixed in paraformaldehyde (Carl Roth, Karlsruhe, Germany) for histological examination. Tendon, peritendineum, subcutis and skin were sutured, and dressings were applied. Post-operative management and pain medication were performed as described above. After taking this biopsy, the hindlimbs were excluded from all further assessments.

24 weeks after MSC injection, the animals were euthanized in general anesthesia using Romifidin 0,06 mg/kg bwt i.v. (Sedivet®, 10 mg/ml, Boehringer Ingelheim Vetmedica GmbH, Ingelheim am Rhein, Germany) and Butorphanol 0,03 mg/kg bwt i.v (Alvegesic®, 10 mg/ml, CP-Pharma Handelsgesellschaft mbH, Burgdorf, Germany) for sedation, Diazepam 0,08 mg/kg bwt i.v. (Diazepam-® Lipuro, 5 mg/ml, B. Braun Melsungen AG, Melsungen, Germany) and Ketamin 2,2 mg/kg bwt i.v (Ursotamin®, 100 mg/ml, Serumwerk Bernburg AG, Bernburg, Germany) for the induction of anesthesia and T61® 6 ml/50 kg bwt i.v. (Intervet Deutschland GmbH, Unterschleißheim, Germany) for euthanasia. The whole metacarpal region of both forelimb SDFT was collected. First, macroscopic evaluation was performed, at which all parameters (swelling, edema, adhesions, redness) were summarized in a score ranging from 0 (normal) to 15 (severe). Afterwards, the tendons were sectioned into 2 cm long pieces, of which the lateral part was fixed in paraformaldehyde for histological examination and the medial part was frozen for gene expression analysis.

### Histology

For histological examination, 3 μm paraffin sections were prepared from the hindlimb biopsies and from the forelimb tendons, stained and assessed as described below. Regarding the forelimb tendons, unless stated otherwise, mean values were calculated from all dissected tendon pieces, representing the whole metacarpal region including proximal and distal healthy areas, for further analysis.

The parameters evaluated in hematoxylin-eosin (HE) staining were polymorphonuclear leukocytes, lymphocytes, macrophages, perivasculitis, necrosis, edema, calcification, collagen disposition, fibrinosis and fiber organization. The microscopic assessment was done by a blinded veterinary pathologist, the score ranging from 0 (normal) to 30 (severe) as described before [28].

Masson’s Trichrome staining was used for evaluation of the percentage of Fuchsin staining corresponding to uninjured or regenerated tendon regions [29]. The percentages of Fuchsin-stained red areas were measured based on whole slide scans obtained in a slide scanner (Pannoramic SCAN, 3DHISTECH, Budapest, Hungary). Red and blue color channels were extracted from the acquired images and respective stained regions were segmented by the Kittler-Illingworth minimum error thresholding method using Mathematica software [30].

Picrosirius red staining and polarized light microscopy were used to assess the occurrence of crimp. Three images from randomly chosen fields of view were obtained per slide using a 10× objective (Olympus BX41 Laboratory microscope equipped with a U-pot drop in polarizer; Olympus GmbH, Hamburg, Germany). The percentages of large crimp (> 15 μm distance) representing healthy, mature crimp, or small and no crimp (< 15 μm distance) representing immature or missing crimp were measured manually by 2 blinded investigators using ImageJ open source software.

DAPI (4′,6-diamidino-2-phenylindole, Carl Roth GmbH + Co. KG, Karlsruhe, Germany) nuclear staining was used to evaluate cellularity based on the nucleated cell fraction. Whole slide scans from the maximum lesion levels were obtained in the slide scanner evaluated in analogy to the Masson’s Trichrome stained slides. The occurrence of nucleated cells represented by the percentage of blue staining (DAPI filter channel) was determined relative to the total area of each section. Similarly, as an indicator for vascularization, the occurrence of erythrocytes was calculated as the percentage of red fluorescence (Rhodamine filter channel), representing erythrocyte autofluorescence, per total section area.

Collagen I immunohistochemical staining was performed on representative sections from the proximal lesion, maximum lesion, and distal lesion for each forelimb tendon and one section of each hindlimb biopsy. Staining was done with anti-collagen I antibody (rabbit polyclonal, Abcam, Cambridge, UK) and a detection kit (EXPOSE Mouse and Rabbit Specific AP, Abcam) according to the manufacturer’s instructions. Two blinded investigators evaluated the absence or presence of collagen I with 0 representing absence, 1 representing mild and 2 representing marked presence of immunostaining.

### Musculoskeletal marker expression

Musculoskeletal marker expression was analyzed by real-time reverse-transcription polymerase chain reaction (RT-PCR) in each forelimb tendon sample piece. Mean values, representing the whole metacarpal region of the tendon, were used for further analysis.

Frozen tendon samples were homogenized, and total RNA was isolated, purified and transcribed into cDNA using the RevertAid H Minus First Strand cDNA Synthesis Kit (Thermo Fisher Scientific). RT-PCR was performed with a 7500 Real Time PCR System (Applied Biosystems, Foster City, USA) as described previously [31]. Primers used are listed in Table 1, ACTB and GAPDH were used as housekeeping genes for relative quantification [32].

**Table 1:** Primers used for RT-PCR analysis of musculoskeletal marker expression

Gene	Primer pair sequences	GenBank accession number	PCR product in bp
ACTB	For: ATCCACGAAACTACCTTCAAC	NM_001081838.1	174
	Rev: CGCAATGATCTTGATCTTCATC		
GAPDH	For: TGGAGAAAGCTGCCAAATACG	NM_001163856.1	309
	Rev: GGCCTTTCTCCTTCTCTTGC		
Collagen 1A2	For: CAACCGGAGATAGAGGACCA	XM_001492939.1	243
	Rev: CAGGTCCTTGGAAACCTTGA		
Collagen 2A1	For: ATTGTAGGACCCAAAGGACC	XM_001496152	199
	Rev: CAGCAAAGTTTCCACCAAGG		
Collagen 3A1	For: AGGGGACCTGGTTACTGCTT	XM_001917620.2	216
	Rev: TCTCTGGGTTGGGACAGTCT		
Decorin	For: ACCCACTGAAGAGCTCAGGA	NM_001081925.2	239
	Rev: GCCATTGTCAACAGCAGAGA		
Tenascin-C	For: CTAGAGTGTCTCACTATCAGG	XM_001916622.2	163
	Rev: CTAGAGTGTCTCACTATCAGG		
Scleraxis	For: TACCTGGGTTTTCTTCTGGTCACT	NM_001105150.1	51
	Rev: TATCAAAGACACAAGATGCCAGC		
Osteopontin	For: TGAAGACCAGTATCCTGATGC	XM_001496152	158
	Rev: GCTGACTTGTTTCCTGACTG		

### Statistical analysis

Using SPSS 20 statistics software (IBM, Ehningen, Germany), Shapiro-Wilk tests were executed to test the hypothesis of normal distribution of data. If this hypothesis was not rejected, paired t-tests were performed to compare MSC-treated tendons with the contralateral controls, otherwise Wilcoxon-tests were performed. *P*-values < 0.05 were considered significant.

## Results

### Clinical and imaging results

Induction of tendon lesions was demonstrable in all tendons. No significant differences between the left and right tendons were observed prior to cell injection, neither clinically nor in imaging. The procedures, including the recovery from tendon biopsies, were tolerated well using the medication described above.

**Fig. 1:**
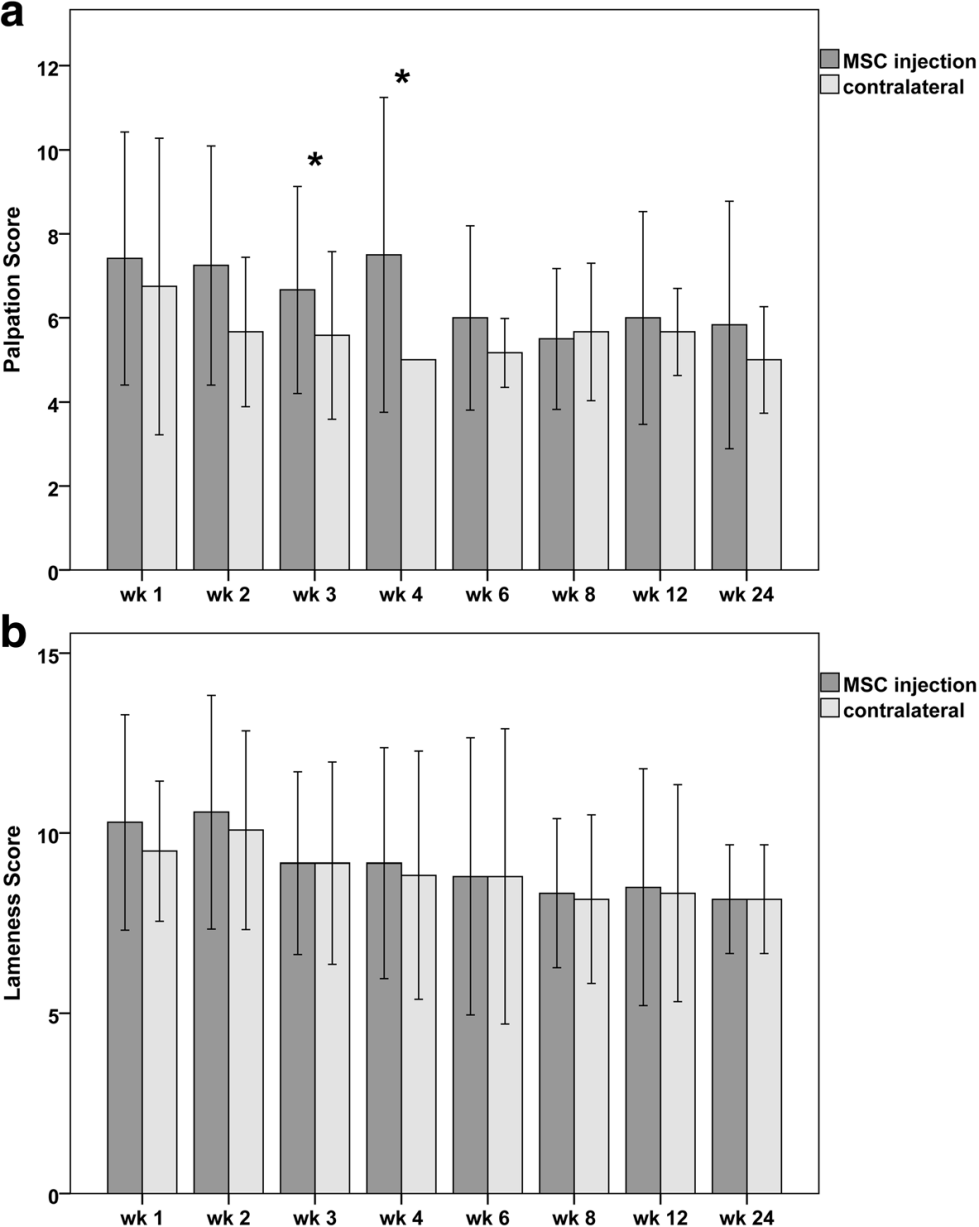
Clinical parameters: Diagrams displaying mean (± 2 SD) values of **a** palpation score and **b** lameness score over the whole follow-up period; stars indicate significant differences between MSC-injected and contralateral tendons (*p* < 0.05); wk.: week post MSC injection

**Fig. 2:**
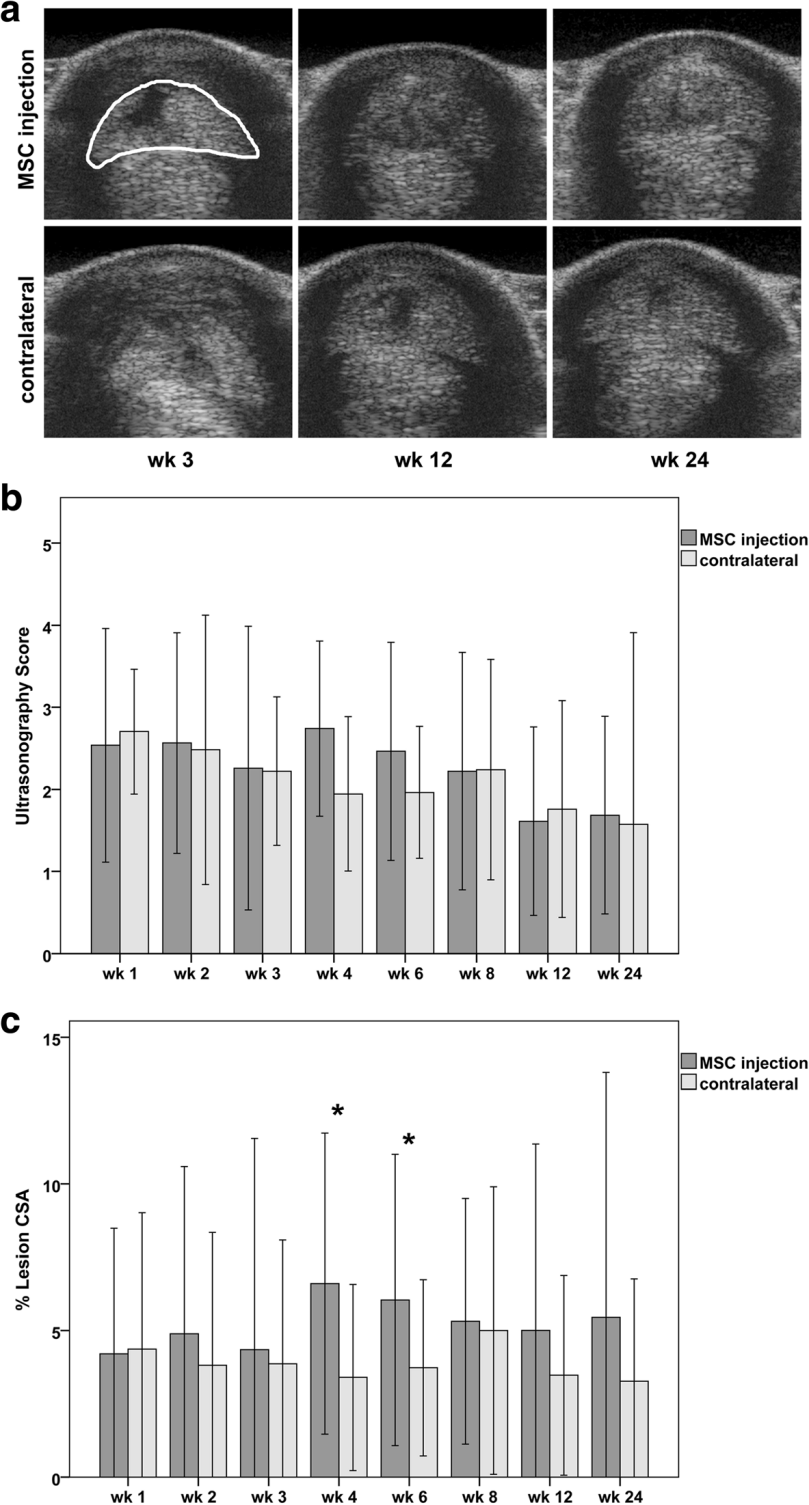
Ultrasonographic parameters: **a** representative transverse images obtained from the MSC-injected and the contralateral superficial digital flexor tendons 3, 12 and 24 weeks after MSC injection; the respective tendon is indicated by the white line in the first upper image; note the hypoechoic (dark) lesions within the tendons, which are decreasing over time. Diagrams displaying mean (± 2 SD) values of **b** ultrasonography score and **c** percentage of the lesion within the cross-sectional area (CSA) of the tendon over the whole follow-up period; stars indicate significant differences between MSC-injected and contralateral tendons (p < 0.05); wk.: week post MSC injection

**Fig. 3:**
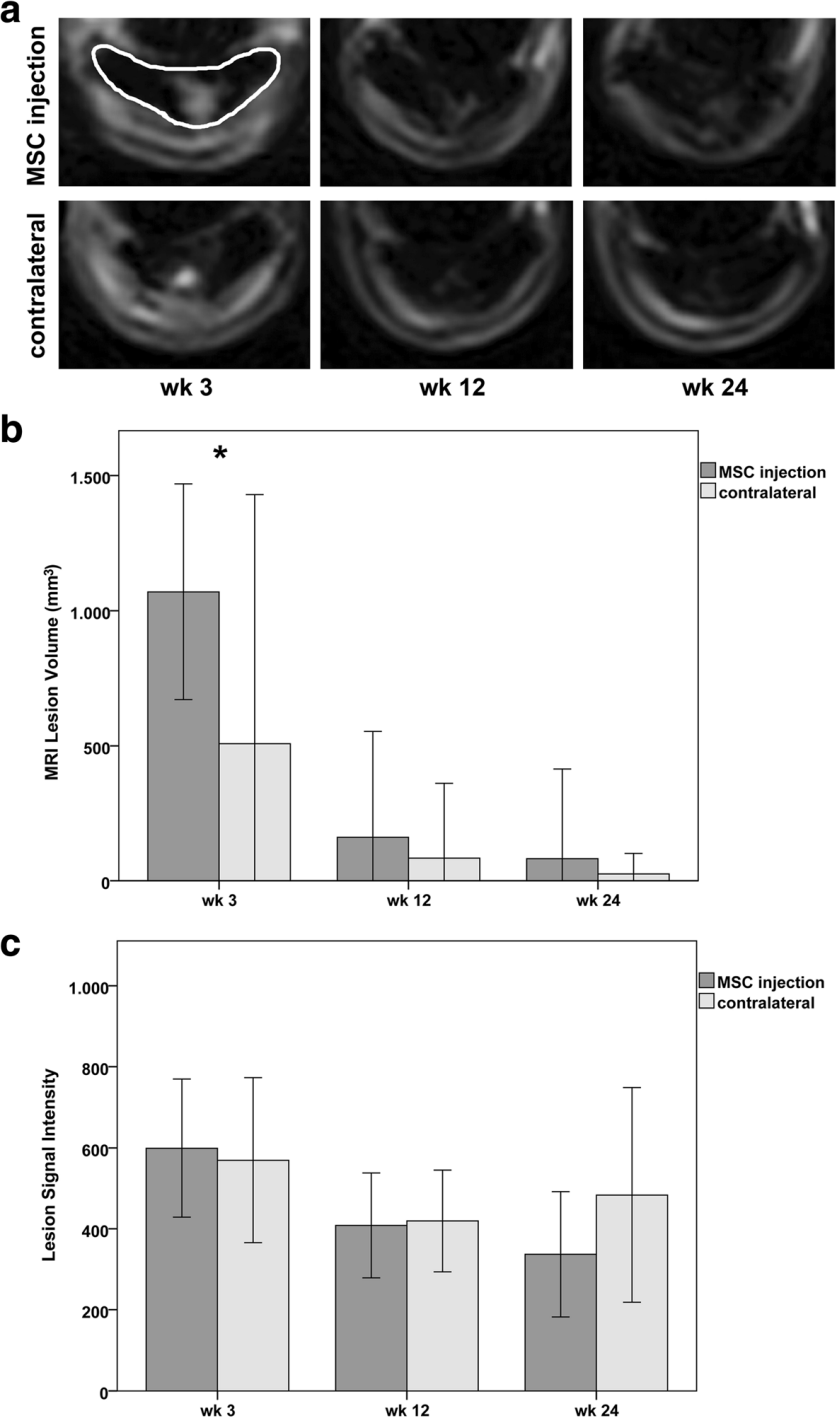
Magnetic resonance imaging parameters: **a** representative transverse T2-weighted images obtained from the MSC-injected and the contralateral superficial digital flexor tendons 3, 12 and 24 weeks after MSC injection; the respective tendon is indicated by the white line in the first upper image; note the lesions within the tendons, displaying high signal intensity at week 3, which is decreasing over time. Diagrams displaying mean (± 2 SD) values of **b** lesion volume determined based on MRI images in mm^3^ and **c** lesion signal intensity obtained from MRI images; star indicates significant difference between MSC-injected and contralateral tendons (p < 0.05); wk.: week post MSC injection; at week 24, instead of *n* = 6 tendons per group, only 2 tendons are shown in the MSC group and 3 in the contralateral control group, due to the fact that the remaining lesions were not detected anymore

During the first 2 weeks after cell injection, no significant differences could be detected between the two groups. However, there was a tendency that the MSC-injected tendons showed prolonged clinical signs of inflammation including swelling, heat, and pain. This became manifest in the palpation scores, which remained higher in the MSC group until week 6, with the difference being significant at weeks 3 (*p* = 0.015) and 4 (*p* = 0.022) (Fig. 1). Correspondingly, as seen in ultrasonography, in the MSC group, the cross-sectional area of the tendon was higher at weeks 3 (*p* = 0.016) and 4 (p = 0.022), the percentage of the tendon lesion was higher at weeks 4 (*p* = 0.042) and 6 (*p* = 0.039), and the mean ultrasonography scores were higher at weeks 4 and 6, although the latter was not significant (Fig. 2). In MRI, lesion volume at week 3 was higher in the MSC group (*p* = 0.02) (Fig. 3).

**Fig. 4:**
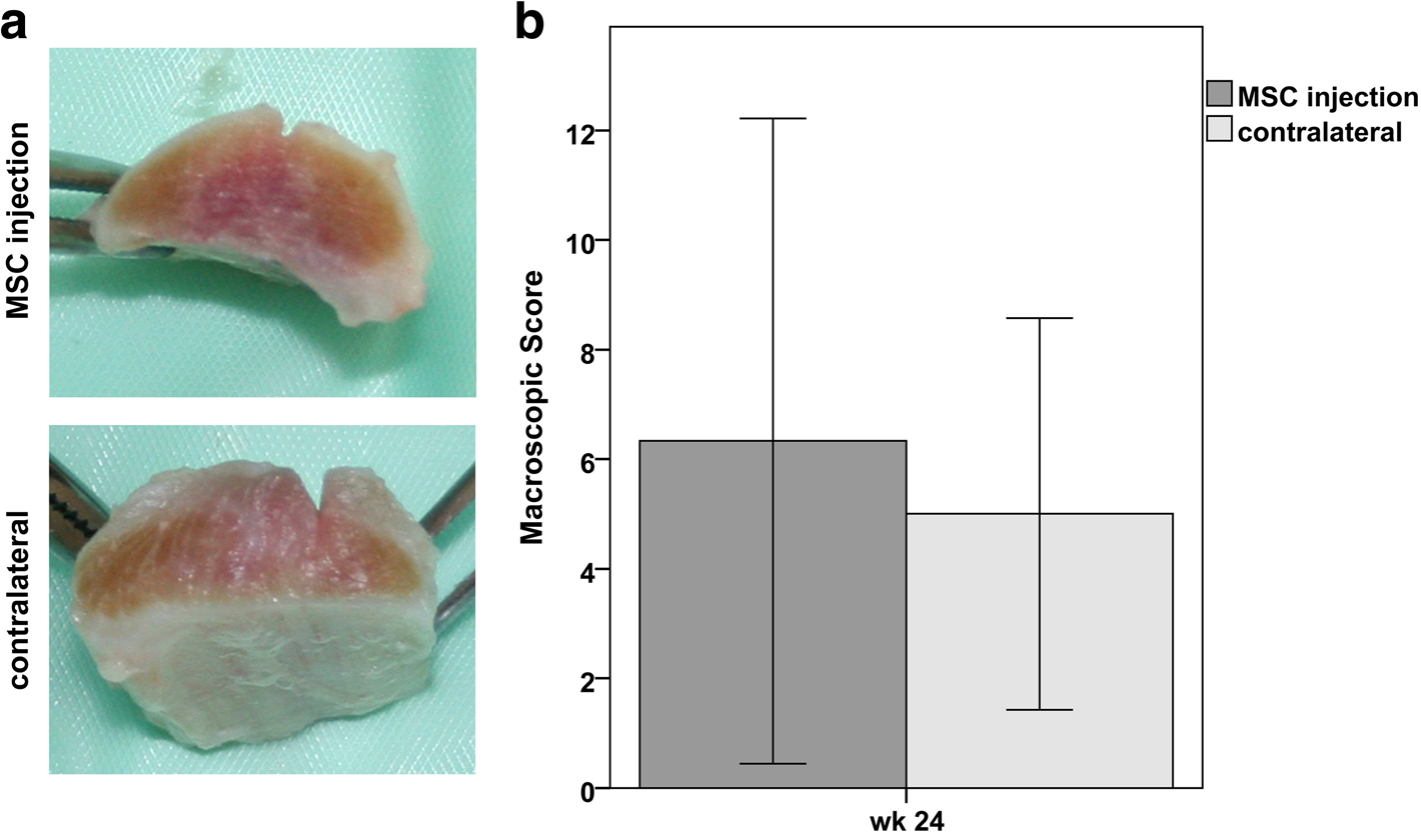
Macroscopic parameters: **a** representative images of the dissected superficial digital flexor tendons at maximum lesion level, displaying reddish and whitish injured areas within the tendon cross-section and **b** diagram displaying mean (± 2 SD) values of score points obtained at macroscopic assessment; wk.: week post MSC injection

However, after the first weeks, the clinical and imaging findings improved and were more similar in both groups. At week 24, based on the T2-weighted MRI image series, the tendon lesions had resolved in 4 out of 6 cases in the MSC group and in 3 out of 6 cases in the control group. In the remaining lesions, the mean lesion signal intensity was lower in the MSC group, suggesting better tendon healing. Further clinical or imaging parameters did not indicate improvement compared to the controls. Correspondingly, macroscopic assessment after euthanasia did not reveal any significant differences (Fig. 4).

### Histology

Histologic findings largely reflected the clinical and imaging findings.

**Fig. 5:**
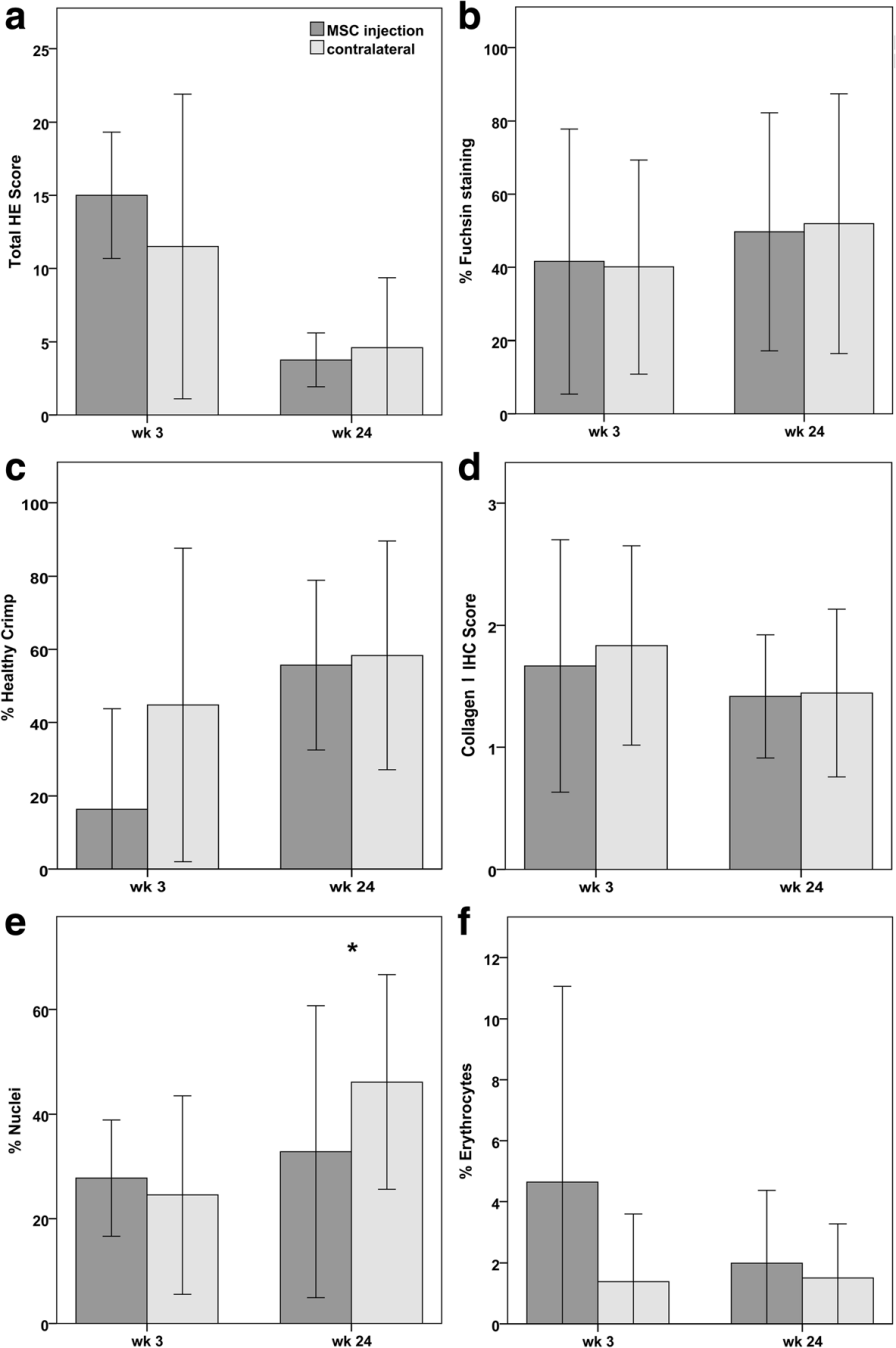
Histology parameters: Diagrams displaying mean (± 2 SD) values of **a** score points obtained at evaluation of hematoxylin-eosin (HE) stained slides, **b** percentage of fuchsin staining representing uninjured or regenerated tendon tissue, **c** percentage of areas displaying healthy crimp, **d** intensity of collagen I immunohistochemical (IHC) staining, **e** percentage of DAPI-stained nuclei indicating cellularity and **f** percentage of erythrocytes indicating vascularization; star indicates significant difference between MSC-injected and contralateral tendons (*p* < 0.05); wk.: week post MSC injection

At week 3, hindlimb biopsies displayed a more frequent occurrence of macrophages and perivasculitis, resulting in a higher HE score, a lower percentage of healthy crimp and a higher percentage of erythrocytes in the MSC group, suggesting increased inflammation and vascularization. However, these differences were not significant, and the percentage of Fuchsin staining, the percentage of nuclei as well as collagen I immunostaining were similar in both groups (Fig. 5).

**Fig. 6:**
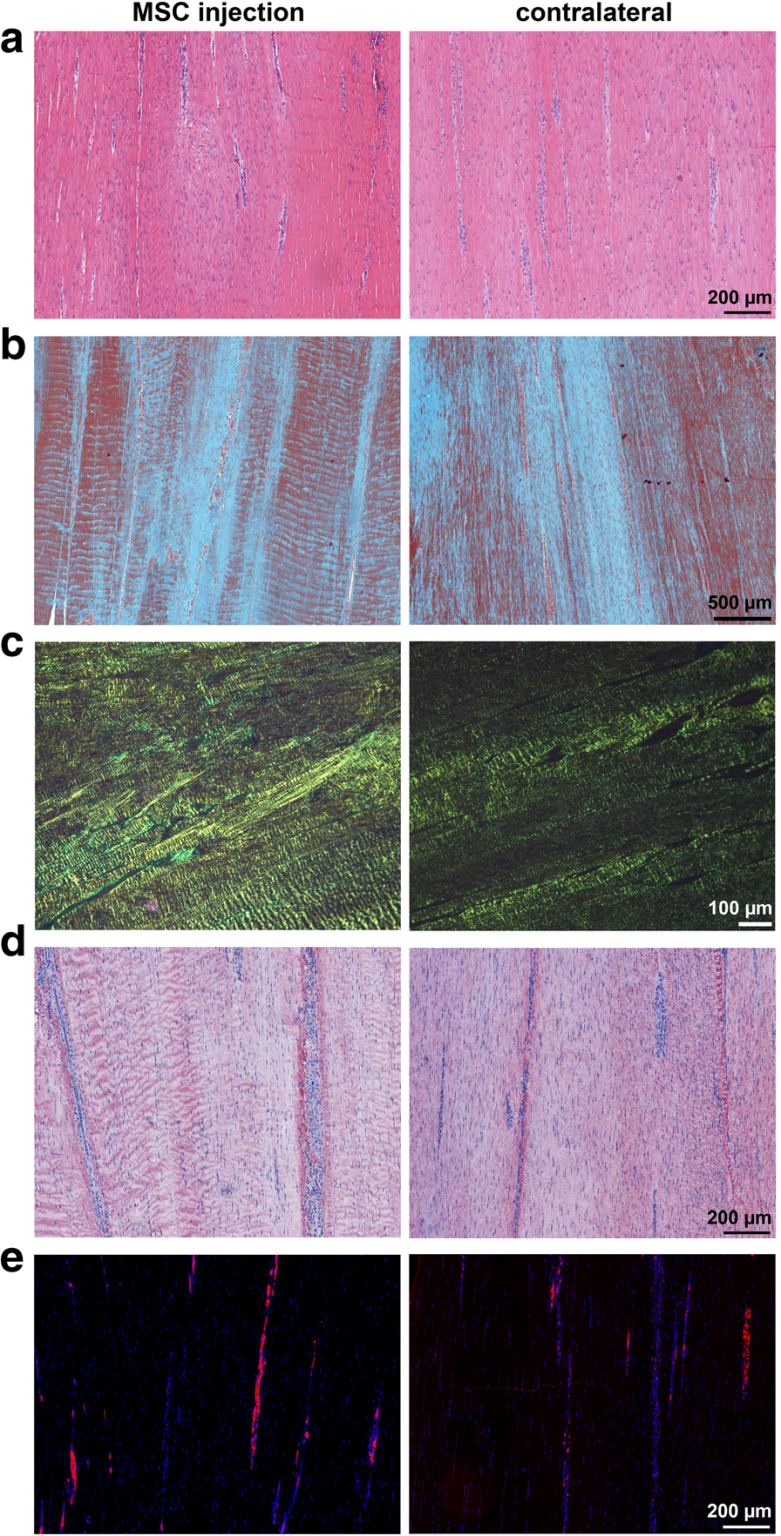
Histology images: Representative images from paraffin sections stained with **a** hematoxylin-eosin, **b** Masson’s Trichrome, **c** Picrosirius red, images obtained using polarized light, **d** anti-collagen I antibody and alkaline phosphatase (red) detection kit and **e** DAPI staining of nuclei (blue), the erythrocytes displaying red autofluorescence

At week 24, such differences did not exist anymore, most stainings and parameters assessed being similar in both groups (Fig. 6). However, the percentage of nuclei was lower in the MSC group (*p* = 0.022), reflecting lower cellularity (Fig. 5).

### Musculoskeletal marker expression

**Fig. 7:**
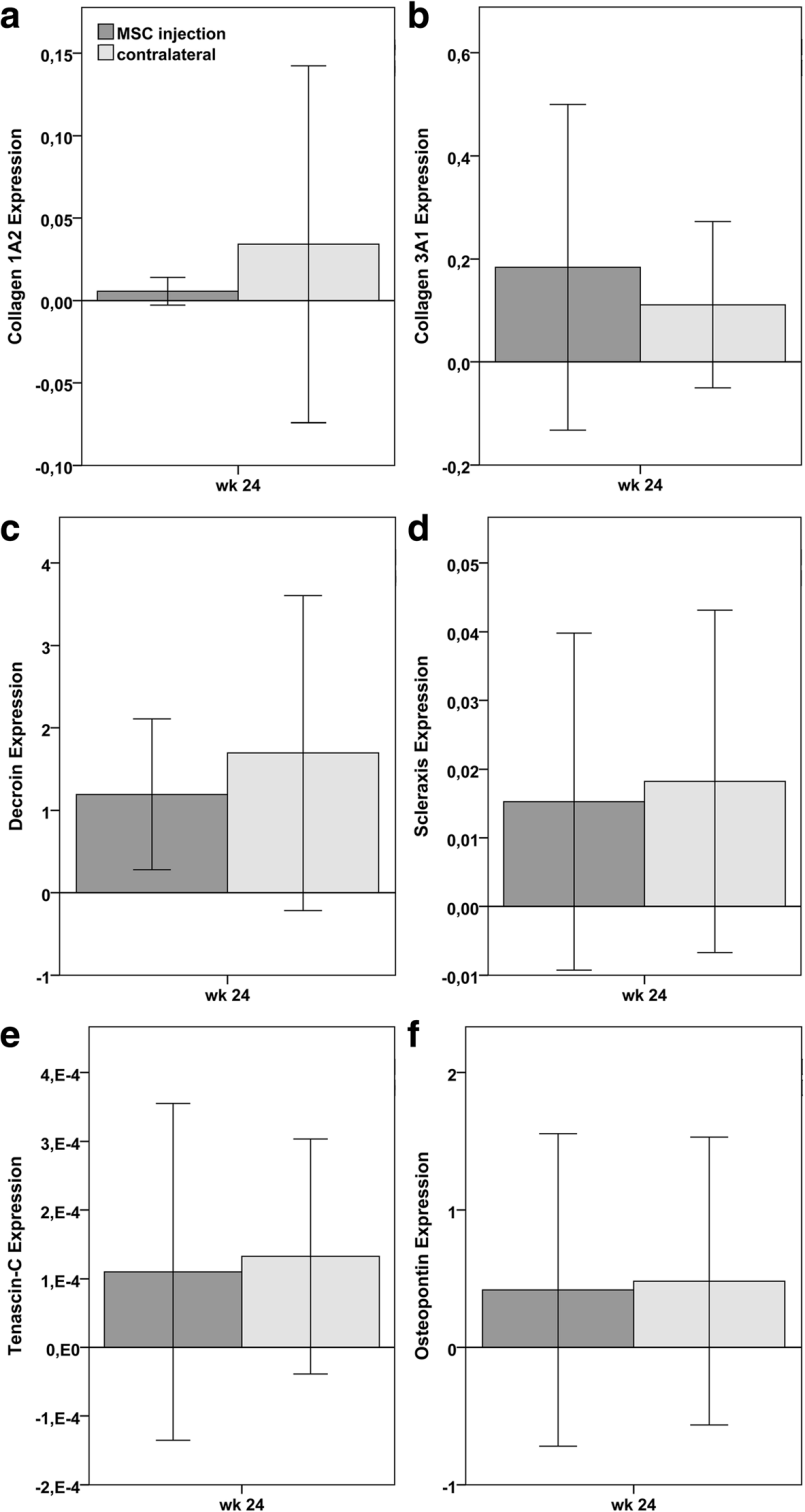
Gene expression: Diagrams displaying mean (± 2 SD) values of relative gene expression of **a** collagen 1A2, **b** collagen 3A1, **c** decorin, **d** scleraxis, **e** tenascin-C and **f** osteopontin, 24 weeks (wk) after MSC injection

All tendon markers including collagen 1A2, collagen 3A1, decorin, tenascin-C and scleraxis, as well as the putative early osteogenic marker osteopontin, were expressed at detectable but variable levels (Fig. 7). Collagen 2A1 was not expressed at detectable levels. No significant differences could be observed between both groups.

## Discussion

Only few differences in tendon healing could be found between MSC and control group at week 24. Differences included that lesion resolution, as seen in T2w MRI, was more advanced and cellularity was lower in MSC-treated tendons at week 24. Furthermore, a transient increase in inflammatory reaction and lesion size was observed in the MSC-treated tendons during the first weeks. However, at week 24, clinical parameters, ultrasound, macroscopic and histological scores as well as musculoskeletal marker expression were not different between treated and control tendons. Based on the fact that MSC had engrafted within the tendon lesions [22] and having designed this study with a relatively long follow-up of 24 weeks, more significant differences between the two groups had been expected based on the previous literature. Yet, the current data fail to provide distinctive evidence to support the hypothesis that MSC improve tendon healing.

The therapeutic approach pursued in the current study was to locally inject adipose-derived MSC 3 weeks after the induction of the tendon lesions. Applying the MSC 3 weeks after induction of the lesion is realistic, because neither in human nor in equine medicine, the patient would receive this autologous therapy within days after the first onset of tendinopathy. Furthermore, the time frame of 3 weeks between induction and treatment should be adequate to support tendon healing, as Crovace et al. found better histological results in the treated groups after choosing an interval of 3 weeks as well [28]. The latter as well as most other studies in horses were conducted with bone marrow-derived MSC [11, 16, 28, 33]. However, the potential of adipose-derived cells is increasingly recognized [34] and adipose-derived cells have also been used for regenerative therapy of tendon lesions with good results [35, 36]. Furthermore, Burk et al. have shown that adipose-derived MSC might be superior to bone marrow-derived MSC regarding their potential to positively influence tendon matrix reorganization [37]. Therefore, the decision to use adipose-derived MSC was favorable, as adipose tissue is easily available with minor surgery in both horses and humans compared to bone marrow, and it remains unlikely that this cell source is the reason for the absence of more significant improvements.

However, the MSC used in the current study had been labeled with SPIO, aiming to track the injected cells and monitor their contribution to tendon healing at the same time. Favorably, these SPIO-labeled MSC have been demonstrated to integrate at the site of intralesional injection [22], in accordance with a similar recent study [38]. It has also been shown in vivo and in vitro that SPIO labeling influences neither stemness nor viability nor proliferation of stem cells [39–42]. In an ovine osteoarthritis model using SPIO-labeled as well as SPIO-negative control MSC, no adverse side effects of SPIO-labeled MSC have been demonstrated [43]. However, current literature also suggests that SPIO influences immune reactions in the surrounding tissue and inhibits endothelial nitric oxide synthase [44, 45]. Therefore, it cannot be excluded that SPIO-labeling of the MSC used in the current study might have compromised their regenerative capacity and/or that released SPIO particles have triggered inflammation.

In this study, the tendon defect was created by a low dose of collagenase injected with a 11 G bone marrow aspiration needle. This large needle creates a mechanical defect in addition to the enzymatical defect. The rationale behind this approach was to combine the advantages of the two most commonly used techniques, collagenase injection and surgical induction, mimicking natural core lesions more closely [46, 47]. This defect is therefore different from those in other studies using higher doses of collagenase and ultrasound-guided injection via smaller needles [28, 33, 48], which is likely to impact on responsiveness to MSC treatment.

Furthermore, the inclusion of adequate controls is crucial. In this study, we chose to use the contralateral tendons as intra-individual controls and to inject these tendons with serum, as serum was used as vehicle to deliver the MSC in the treatment group. The vehicle for MSC delivery should ideally maintain MSC viability prior to injection and support their regenerative effect. While for bone marrow MSC, bone marrow supernatant is most commonly used, serum is a good alternative for other MSC sources. However, saline or phosphate buffered saline (PBS) have frequently been chosen for injection into the control tendons [11, 33, 36], which is not indicative as to whether any observed effect is due to MSC treatment or the delivery vehicle. In this line, Geburek et al. used autologous conditioned serum (ACS) without MSC for treatment of naturally occurring tendinopathy and reported on significant reduction of lameness and swelling in the ACS group compared to saline [17]. This suggests that serum itself may have beneficial effects on tendon healing. Consequently, differences found in studies using PBS or saline for control injections but serum or bone marrow supernatant as MSC delivery vehicle [11, 33, 36] cannot without doubt be attributed solely to the MSC. This could partly explain that fewer beneficial effects were detected in the current study, in which serum was used as a more rigorous control regarding MSC efficacy.

The use of the contralateral tendon of the same animal as control limits interference of inter-individual differences with the results, reduces the numbers of animals required, and has been described by several groups [28, 33, 38]. However, MSC could enter the blood circulation after injection and migrate to other sites of injury or inflammation as well, e.g. the contralateral defect, a process referred to as homing [49, 50]. MSC have been shown to circulate in peripheral blood after intralesional injection but had not been found in untreated lesions so far [51, 52]. Yet, in the previously published part of the current study, MSC could be shown to be present in the contralateral tendon lesions as well, albeit in small numbers [22]. Based on that, the effect of MSC distribution after intralesional injection might have been underestimated when the current study was designed. However, we still consider a strong impact on the results as unlikely, as significantly fewer cells were found in the control lesions compared to the treated lesions.

Regarding sample and data analysis, while other studies used only the maximum lesion zone for imaging assessment and the macroscopically affected areas of the tendon for histological examination [28, 33, 48], we analyzed the whole metacarpal area of the tendon. This may have hampered the detection of differences potentially existing at the maximum lesion sites only. However, this method is more rigorous as it takes the whole longitudinal extension of the defect into account, and there is no potential bias as subjective identification of maximum lesion site is not required.

Possibly elucidating which of the discussed factors and differences between studies might most likely explain the outcome, two previous studies are particularly interesting for comparison. A recent carefully designed study, investigating the effect of autologous adipose-derived MSC suspended in inactivated serum in surgically created tendon lesions in the horse, over a follow-up period of 22 weeks, also revealed few improvements. The authors did not find significant differences in histology, biochemical or biomechanical parameters between MSC-treated lesions and serum-injected contralateral control tendon lesions, although hydroxylysylpyridinoline content in the MSC group was closer to that of healthy tendon tissue, potentially indicating better crosslinking [38]. In a different study, the effect of bone marrow-MSC suspended in bone marrow supernatant for treatment of naturally occurring tendon lesions was investigated. After a follow-up of 6 months post injection, treatment outcome was favourable, with improved tissue structure, lower cellularity, vascularity, water and glycosaminoglycan content as well as matrix metalloproteinase-13 activity. While it should be considered that controls were based on saline injections, this study stands out because naturally occurring tendon lesions were used, instead of artificially creating them [11].

Considering the outcome of these two studies in line with the current results, it appears most likely that MSC are not capable to repair mechanically induced tendon lesions, potentially due to the associated loss of tissue that cannot be replaced adequately over a follow-up period of 22 or 24 weeks, respectively. The choice of the animal model is crucial in clinical translation and can strongly impact on the results of preclinical studies. Although we aimed to overcome the limitations of collagenase-based and surgical lesion induction by combining mild approaches of both techniques, the current model might still not reflect naturally occurring tendinopathy. Therefore, taking advantage of the fact that horses are equally prone to natural tendon disease as humans, to use them as natural models as reported by Smith et al. still represents the most reliable option- albeit not without challenges [11]. With equal importance, it remains possible that MSC are not significantly more effective than delivery vehicles such as bone marrow supernatant or serum, even if inactivated, indicating that this fundamental question has yet to be answered.

Yet, we observed significant differences during the early healing phase between weeks 3 and 6, at which clinical assessment and imaging suggested a stronger but transient inflammatory reaction in MSC-treated tendons. Although based on the current data, it cannot be excluded that SPIO might have induced this inflammatory response, our results correspond to a previous study, in which a transient increase in vascularization after injection of MSC was observed, while the latter were not labeled with SPIO [35]. This could be part of the immunomodulatory effects of MSC, which could be important for debridement and lesion resolution and to prevent fibrotic repair, thus there is reason to consider that this transient inflammatory reaction could be beneficial [53]. However, except for the lower cellularity of treated tendons, no distinctive evidence to support this hypothesis was found in this study, thus further studies need to elucidate the immunomodulatory effects of MSC in tendon healing.

## Conclusions

Intralesional injection of adipose-derived MSC led only to minor improvements of tendon healing within the observed time frame of 24 weeks, indicating that MSC were not capable of repairing the mechanically disrupted tissue within the tendon lesions during the observed period. Future studies, ideally based on using naturally occurring tendon lesions as a model and using the adequate controls, still have to answer the fundamental question whether the effect of MSC is superior to that of biologically active body fluids such as serum. However, we observed that MSC induced a transient inflammation during early healing, followed by reduced cellularity in treated tendons at week 24, which suggests a modulatory effect warranting further investigations.

## Abbreviations

ACS: Autologous Conditioned Serum
ACTB: Actin Beta
BMBF: German Federal Ministry of Education and Research
cDNA: Complementary Deoxyribonucleic Acid
cm: Centimeters
DAPI: 4′,6-diamidino-2-phenylindole
DMEM: Dulbecco’s Modified Eagle’s Medium
EMA: European Medicines Agency
FBS: Fetal Bovine Serum
FDA: Food and Drug Administration
Fe: Iron (Ferrum)
G: Gauge
GAPDH: Glyceraldehyde 3-phosphate dehydrogenase
HE: Hematoxylin-Eosin
IHC: Immunohistochemical
IU: International Unit
mg/kg bwt: Milligrams per Kilogram body weight
ml: Milliliters
mm: Millimeters
MRI: Magnetic Resonance Imaging
MSC: Mesenchymal stromal cells
PBS: Phosphate Buffered Saline
RNA: Ribonucleic Acid
RT-PCR: Real-time reverse-transcription polymerase chain reaction
SD: Standard Deviation
SDFT: Superficial Digital Flexor Tendon
SMWK: Saxon Ministry of Science and the Fine Arts
SPIO: Superparamagnetic Iron Oxide
T2w: T2-weighted
US: Ultrasound
wk: Week
